# Endoscopic Assisted Percutaneous Fixation of Anterior Inferior Iliac Spine Avulsion Fracture, Surgical Technique

**DOI:** 10.1007/s43465-024-01107-5

**Published:** 2024-02-22

**Authors:** Alessandro Aprato, Andrea Donis, Michele Reboli, Jacopo Vittori, Davide Carlo Secco, Alessandro Massè

**Affiliations:** School of Medicine, University of Turin, Turin, Italy

**Keywords:** Endoscopic reduction and fixation, Anterior inferior iliac spine, Avulsion fracture, Adolescent athletes, Sport injury

## Abstract

Avulsion fractures of the anterior inferior iliac spine rarely occur in adolescent athletes during rectus femoris contractions or eccentric muscle lengthening while the growth plate is still open. Currently, there are no official guidelines in the literature on the treatment indications of this type of fracture or the type of surgical technique to be used. Nowadays, young and athletic patients desire a quick return to their previous activities, which makes surgical treatment a reasonable choice. Open reduction and internal fixation with an anterior approach are usually recommended when the avulsion fragment has more than 1.5–2 cm displacement on plain radiographs. However, ORIF is associated with a higher risk of heterotopic ossifications and increases the risk of damage to the LFCN. An endoscopic technique was designed to reduce these complications. This technical note describes a procedure of percutaneous fixation to AIIS through 3 endoscopic portals that could potentially minimize complications associated with an open surgical dissection, allowing anatomic reduction under direct visualization.

## Introduction

In orthopedic clinical practice, anterior inferior iliac spine avulsion fractures (AIIS) are rare injuries that occur mainly in adolescent athletes due to severe contracture of the rectus femoris while the hip is hyperextended and the knee is flexed. Currently, there are no official guidelines in the literature on the indications for treatment of this type of fracture or the type of surgical technique to be used [1]. Since there are no specific indications for the treatment of AIIS avulsion fractures, only general indications for the treatment of pelvic avulsion fractures can be found in the literature. This article aims to describe an endoscopic assisted percutaneous osteosynthesis of AIIS, with the support of intraoperative illustrations, and to share several advantages and potential risks related to this surgical procedure.

## Indications and Contraindications

The indication for the AIIS avulsion fracture endoscopic assisted reduction and fixation was a single grossly displaced (> 1.5 cm in anterior–posterior pelvic radiograph) fragment in highly active athlete who needed a fast return to play.

Contraindications were nondisplaced/minimally displaced fractures, comminute fractures in which the use of a screw was not recommended, sedentary patients, and local infections.

## Surgical Technique

### Patient Positioning and Operative Room Setting

Patient under general anaesthesia, supine on the radiolucent bed and without traction, mobile C-arm image amplifier, and arthroscopic column contralateral to the side of the fracture.

### Surgical Field Setting

Thorough skin disinfection with 2% Chlorhexidine solution, four sterile disposable drapes to the border: proximally the iliac crest, anteriorly at the level of the medial third of the groin, distally at the middle third of the thigh, and posteriorly behind the greater trochanter. The lower limb to be operated on must remain free to be eventually flexed during the surgery.

## Endoscopic Portals (Fig. 1)

**Fig. 1:**
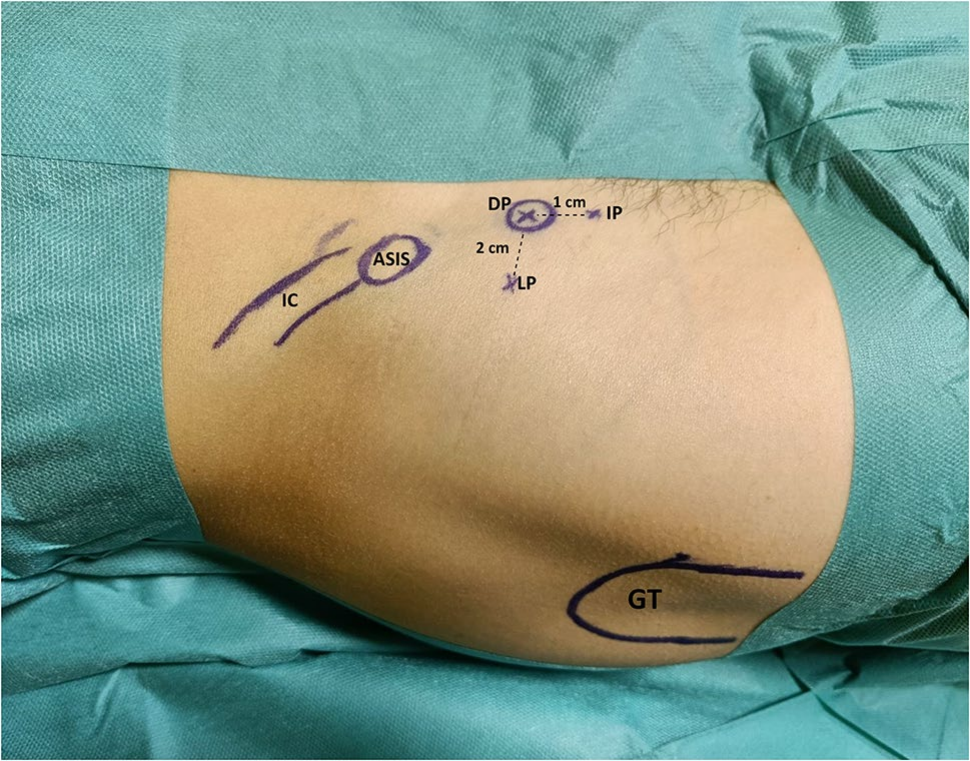
Arthroscopic portals and lanmarks. IC: iliac crest, ASIS: anterior superior iliac spine, GT: greater trochanter, DP: direct portal, directly on the anterior inferior iliac spine, IP: inferior portal, 1 cm inferiorly to AIIS, LP: lateral portal, 2 cm lateral to AIIS

Under C-arm image amplifier controls, three endoscopic portals are identified:

- Lateral Portal (LP): 2 cm lateral to the AIIS;
- Direct Portal (DP): directly on the AIIS;
- Inferior Portal (IP): 1 cm inferior to the AIIS.

## Surgical Instruments

- Arthroscope with 30° optic
- Nitinol wires
- Radiofrequency
- Shaver
- K-Wire 2 mm diameter
- 1 partially threaded screw 4.5 mm diameter

## Surgical Procedure

On a radiolucent bed, the limb to be operated on is left free so that the hip can be flexed during surgery if necessary. Through intraoperative x-rays, the AIIS is identified so the three endoscopic portals can be located: LP, DP, and IP. (Fig. 1).

**Fig. 2:**
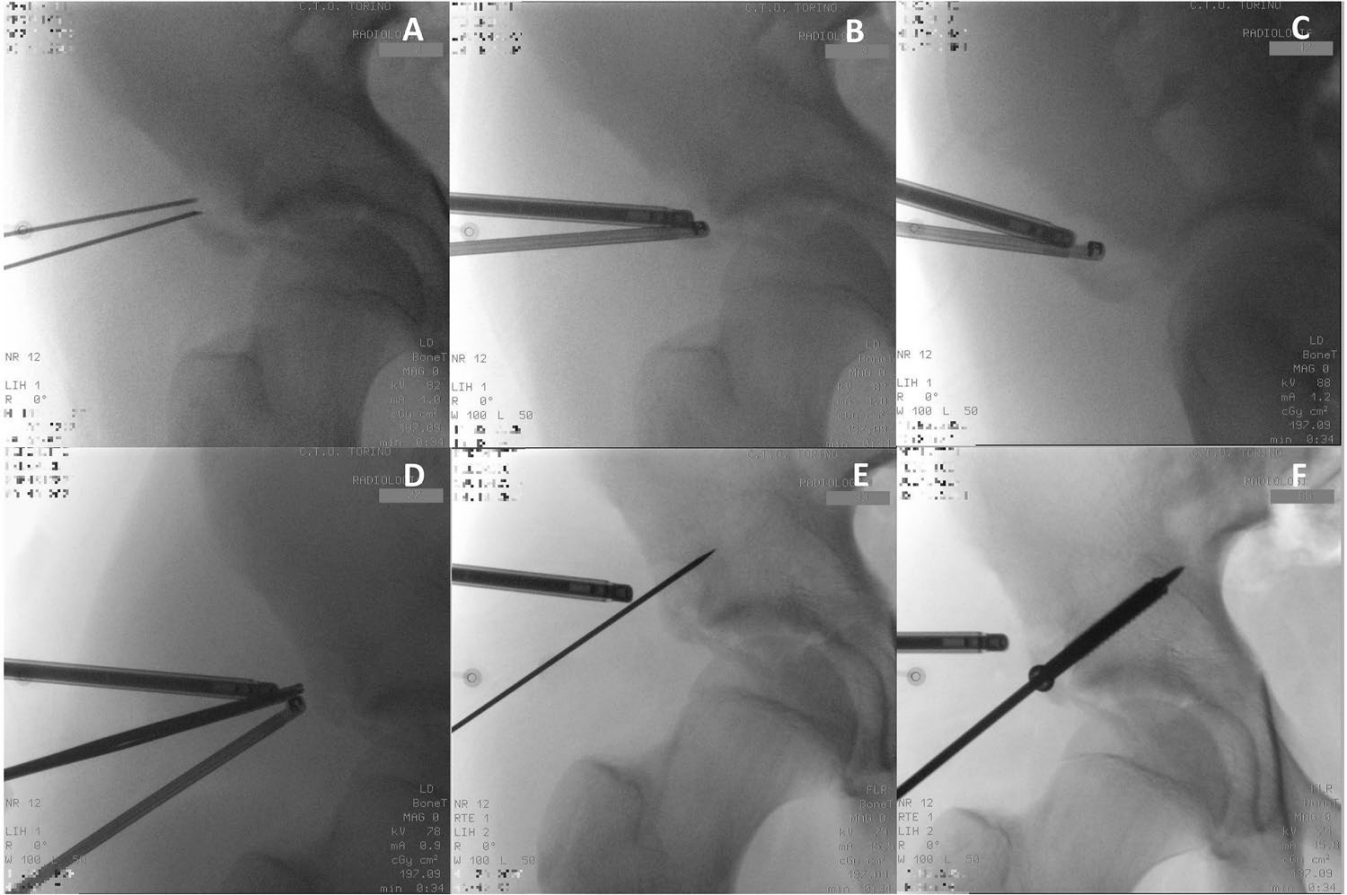
Amplioscopic views, **A** and **B** nithinol wires and optic and radiofrequency insertion through DP and LP; **C** mobilization and debridement of the fragment, **D** reduction; **E** K-wire temporary fixation; **F** screw insertion

Starting from the LP and DP portals, nitinol wires are introduced, and then the 30° optic and the radiofrequency are placed, respectively (Fig. 2A, B). The water pump is activated until a pressure of 40 mmHg is reached. Approaching the LP it is crucial to avoid being too lateral to the AIIS as there is a risk of entering the territory of the LFCN [2]; in fact, it emerges under the inguinal ligament, just medial to the ASIS and descends along the surface of the sartorius muscle, in case of injury there is a risk of causing paresthetic meralgia. On the medial side, femoral vascular-nervous structures are at risk, so care must be taken to locate the DP and IP portals.

**Fig. 3:**
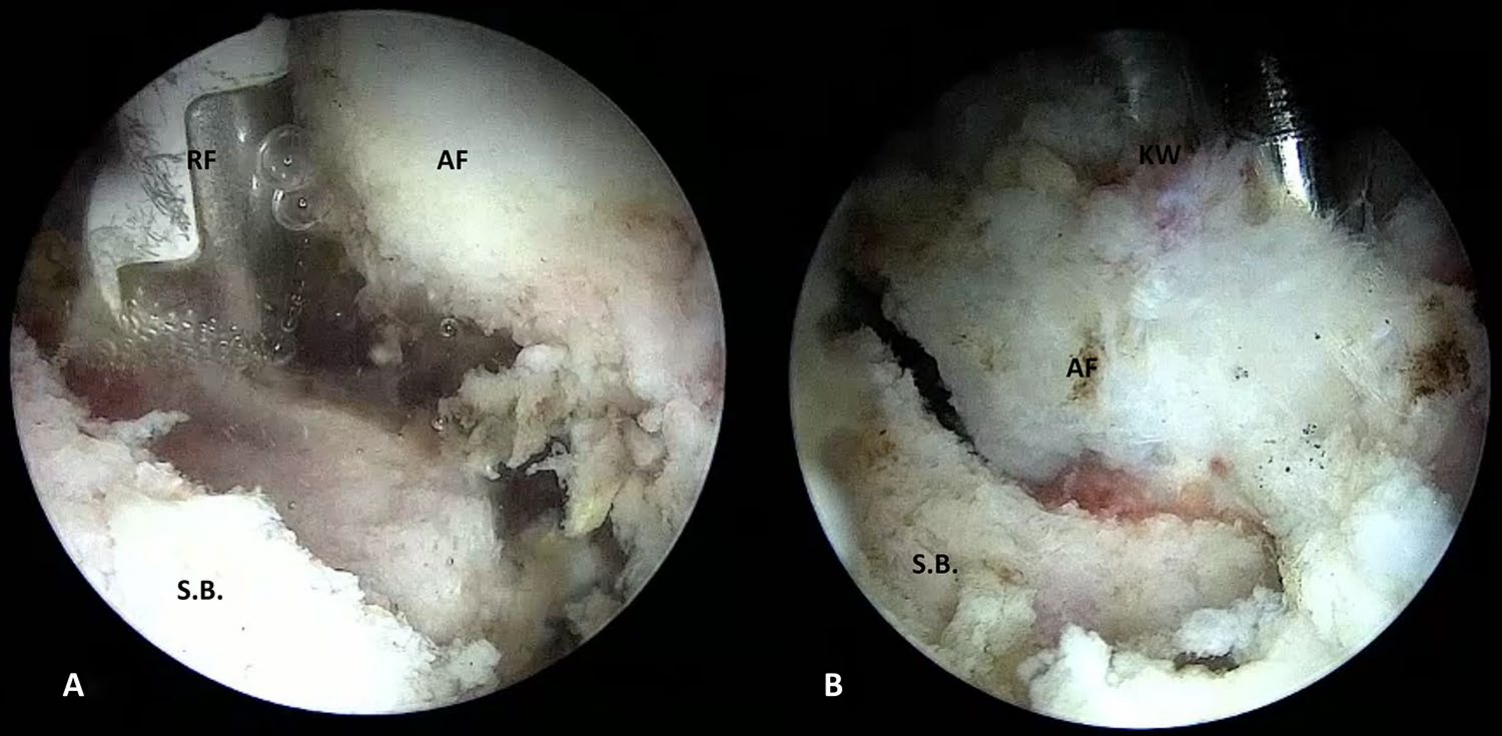
Endoscopic views, **A** interfragmentary debridement with radiofrequency (RF); **B** K-wire temporary fixation. AF: avulsed fragment, SB: AIIS base, KW: K-wire

By endoscopic vision, the fracture is identified; through the use of a shaver and radiofrequency, an accurate debridement of the fracture's interface is performed, allowing it to be mobilized (Figs. 2C, 3A).

Through the IP, a switching stick or a shaver is inserted, and it can be used for the reduction maneuver, with the aim of reducing the fragment as anatomically as possible. If there is difficulty in retracting the avulsed fragment, flexion of the hip can be helpful, detending the rectus femoris muscle (Fig. 2D).

Once anatomical reduction is achieved, a 2 mm k-wire is inserted perpendicular to the fracture by the same portal, and an intraoperative x-ray check is necessary to confirm adequate temporary reduction (Figs. 2E, 3B).

**Fig. 4:**
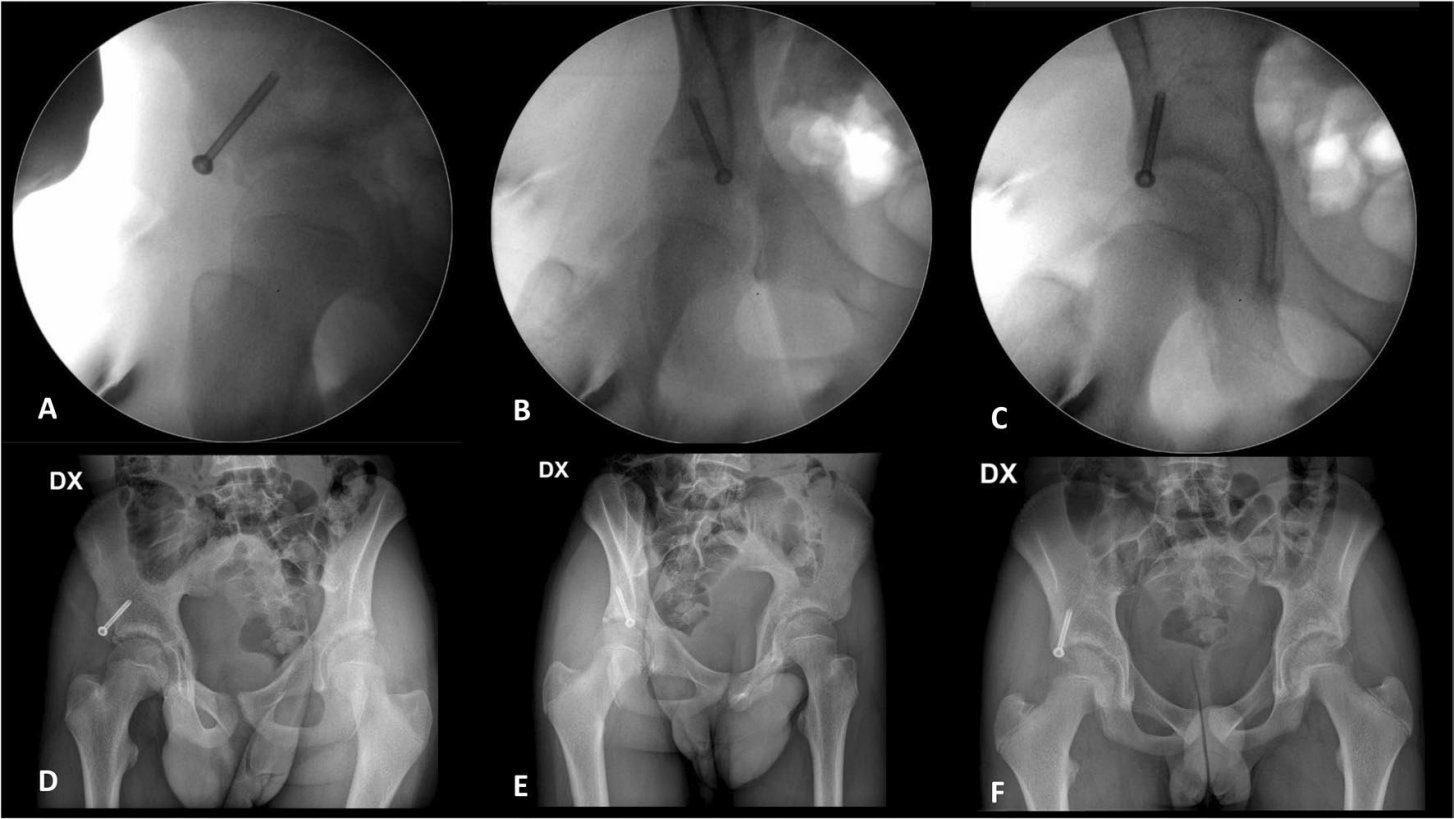
**A**–**C** fluoroscopic final controls; **D**–**F** XR postoperative controls

Finally, the definitive fixation can be performed with a partially threaded cannulated interfragmentary screw 4.5 mm in diameter inserted by the IP. (Fig. 2G). The correct anatomical reduction and screw insertion are ensured through x-rays control in Judet's iliac wing projection (Fig. 4A–C).

The skin is closed with resorbable wires and single stitches, and medication is applied.

The final result is evaluated with postoperative x-ray in Anterior–Posterior (AP), obturator, and Judet's wing projection (Fig. 4D–F).

## Discussion

**Fig. 5:**
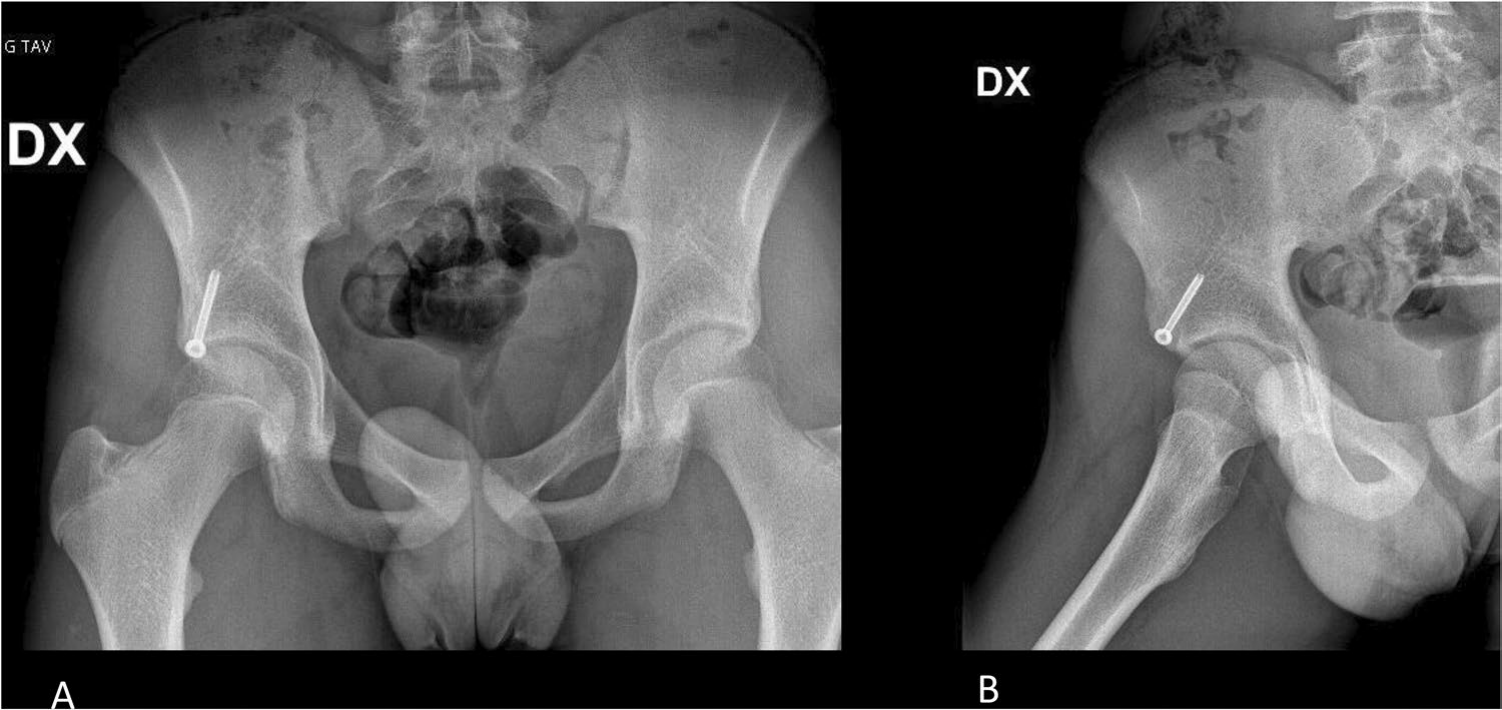
**A**, **B** One year follow up x-rays. **A** AP view, **B** Oblique view

The anterior inferior iliac spine (AIIS) is a frequent site of avulsion fracture in the pelvis in teenage athletes where the hormonally induced muscle strengthening coexists with secondary ossification at the apophyses [3]. Most pelvic avulsion fractures, including AIIS, are treated successfully nonoperatively, using analgesics, bed rest, immobilization of the affected muscle group, physical rehabilitation, and crutches for at least 3–6 weeks post-injury [1, 6–8]. Although, avulsion fractures of the pelvis have been biologically assimilated to epiphyseal fractures and are naturally prone to bone healing [4], repetitive traumatism, due to muscle traction over the avulsed fragment, often leads to hypertrophic healing, bulking, and appearance that may mimic bone tumours, especially when a delayed diagnosis or no supervised conservative treatment is made [5]. Open reduction and internal fixation are usually recommended when the avulsion fractures have a displacement of more than 1.5–2 cm on plain radiographs and in athletes who need a faster return to play [1, 3]. In fact, current evidence suggests that patients treated surgically return to sport more than those receiving conservative treatment (92% vs. 80%), with a higher rate of excellent outcomes when the displacement on plain radiographs is major than 1.5 cm [1]. As noted by Hetsroni et al. [9] in 2013, the AIIS morphology is a potential mechanical contributor to a hip impingement in young patients. Different authors have recently demonstrated how AIIS avulsion fracture could lead to hip dysfunction due to abnormal contact between the femoral neck and the abnormal supra-acetabular region [10–12], a condition known as sub-spine femoral-acetabular impingement [13]. In this context, anatomic reduction and internal fixation of the AIIS fragment could potentially lead to direct bone healing without callus formation and with less post-traumatic deformation. Although open reduction and internal fixation doesn’t seem to expose a significant risk to conservative treatment overall, it has been associated with a higher risk of heterotopic ossifications [1]. Moreover, the use of an anterior surgical approach increases the risk of damage at the LFCN, while careful localization and protection of the nerve may be particularly difficult due to its variable anatomy [14]. This technical note is the first description of AIIS avulsion fracture endoscopic assisted reduction and fixation. The proposed approach might be considered a less invasive surgical technique, conceived to prevent some of the complications associated to open reduction and internal fixation of displaced AIIS avulsion fractures. This surgical technique, performed by an expert arthroscopic hip surgeon, could potentially minimize complications associated with an open surgical dissection, allowing anatomic reduction under direct visualization and percutaneous fixation. The minimal dissection of the soft tissues creates a more favorable environment for wound and fracture healing. The authors believe that this technique is facilitated by the relatively large size (approx. 1.5 cm) of the avulsion fragment as smaller fragments may be more difficult to identify endoscopically. Our initial clinical and radiographic results are promising, with no complications referable to a lateral femoral cutaneous nerve injury or associated with the surgical wound. An adequate reduction was achieved according to the postoperative radiographs (anteroposterior, obturator, and iliac views), and no delayed unions, non-unions, or hypertrophic malunions were observed. The patient was able to start early range of motion and progressive weight bearing without pain on the first day after the surgery**. **At one year follow up, the patient has returned to his preinjury level of activities without limitations. Range of motion was full and comparable to the contralateral hip with no signs of paresthetic meralgia in the territory of LFCN. One year follow-up x-rays **(**Fig. 5) show bone healing without callus formation and no heterotopic ossifications. Hardware removal was not necessary. Table 1 shows the advantages and risks of endoscopically assisted reduction and fixation of AIIS avulsion fracture. Further limitations of the proposed technique are the costs associated with the arthroscopic instrumentation, a possible extension of surgical time and the demand for excellent arthroscopic skills. Studies comparing different techniques, as well as investigations with a larger population, are needed.

**Table 1:** Advantages and risks of endoscopically assisted reduction and fixation of anterior inferior iliac spine avulsion fracture

Advantages	Risks
1. Small incisions and better cosmetic outcome	1. Malreduction
2. Minimal soft-tissue trauma	2. Malunion
3. Anatomical fracture reduction under endoscopic guidance	3. Nonunion
4. Possible assessment and treatment of concomitant intra-articular pathology	4. Nerves and vascular injuries
5. Possible reduction of LCFN injury risk	
6. Possible reduction of heterotopic ossifications risk	

## Abbreviations

AIIS: Anterior inferior iliac spine
ASIS: Anterior superior iliac spine
DP: Direct portal
LP: Lateral portal
IP: Inferior portal
LFCN: Lateral femoro-cutaneous nerve
AP: Anterior–posterior view

## References

[1] Eberbach H, Hohloch L, Feucht MJ, Konstantinidis L, Südkamp NP, Zwingmann J (2017). Operative versus conservative treatment of apophyseal avulsion fractures of the pelvis in the adolescents: A systematical review with meta-analysis of clinical outcome and return to sports. BMC Musculoskeletal Disorders.

[2] Aprato A, Giachino M, Masse A (2016). Arthroscopic approach and anatomy of the hip. Muscles Ligaments Tendons Journal.

[3] McKinney BI, Nelson C, Carrion W (2009). Apophyseal avulsion fractures of the hip and pelvis. Orthopedics.

[4] Metzmaker JN, Pappas AM (1985). Avulsion fractures of the pelvis. American Journal of Sports Medicine.

[5] Lambrechts MJ, Gray AD, Hoernschemeyer DG, Gupta SK (2020). Hip impingement after anterior inferior iliac spine avulsion fractures: A case report with review of the literature. Case Reports in Orthopedics.

[6] Kishta W, Lane TS, El-Hawary R (2014). Sequential ipsilateral avulsion of the anterior inferior iliac spine and the anterior superior iliac spine in an adolescent patient: A case report. JBJS Case Connect.

[7] Oldenburg FP, Smith MV, Thompson GH (2009). Simultaneous ipsilateral avulsion of the anterior superior and anterior inferior iliac spines in an adolescent. Journal of Pediatric Orthopaedics.

[8] Weel H, Joosten AJP, van Bergen CJA (2022). Apophyseal avulsion of the rectus femoris tendon origin in adolescent soccer players. Children.

[9] Hetsroni I, Poultsides L, Bedi A, Larson CM, Kelly BT (2013). Anterior inferior iliac spine morphology correlates with hip range of motion: A classification system and dynamic model hip. Clinical Orthopaedics and Related Research.

[10] Shibahara M, Ohnishi Y, Honda E, Matsuda DK, Uchida S (2017). Arthroscopic treatment of a displaced nonunion of the anterior inferior iliac spine causing extra-articular impingement. Orthopedics.

[11] Nakano N, Lisenda L, Khanduja V (2018). Arthroscopic excision of heterotopic ossification in the rectus femoris muscle causing extra-articular anterior hip impingement. Sicot-Journal.

[12] Hetsroni I, Larson CM, Dela Torre K, Zbeda RM, Magennis E, Kelly BT (2012). Anterior inferior iliac spine deformity as an extra-articular source for hip impingement: A series of 10 patients treated with arthroscopic decompression. Arthroscopy: The Journal of Arthroscopic & Related Surgery.

[13] Larson CM, Kelly BT, Stone RM (2011). Making a case for anterior inferior iliac spine/subspine hip impingement: Three representative case reports and proposed concept. Arthroscopy: The Journal of Arthroscopic & Related Surgery.

[14] Tomaszewski KA, Popieluszko P, Henry BM, Roy J, Sanna B, Kijek MR, Walocha JA (2016). The surgical anatomy of the lateral femoral cutaneous nerve in the inguinal region: A meta-analysis. Hernia.

